# Biomass estimation of cultivated red algae *Pyropia* using unmanned aerial platform based multispectral imaging

**DOI:** 10.1186/s13007-021-00711-y

**Published:** 2021-02-04

**Authors:** Shuai Che, Guoying Du, Ning Wang, Kun He, Zhaolan Mo, Bin Sun, Yu Chen, Yifei Cao, Junhao Wang, Yunxiang Mao

**Affiliations:** 1 Key Laboratory of Marine Genetics and Breeding (Ministry of Education), College of Marine Life Sciences, Ocean University of China, Qingdao, 266003 People’s Republic of China; 2 Key Laboratory of Utilization and Conservation of Tropical Marine Bioresource (Ministry of Education), College of Fisheries and Life Science, Hainan Tropical Ocean University, Sanya, 572022 People’s Republic of China; 3 Laboratory for Marine Biology and Biotechnology, Pilot National Laboratory for Marine Science and Technology (Qingdao), Qingdao, 266000 People’s Republic of China; 4 Xi’ an Ecotech Spectral Imaging and Eco-drone Remote Sensing Research Center Co., Ltd., Xi’ an, 710000 People’s Republic of China

**Keywords:** *Pyropia*, Biomass estimation, Unmanned aerial platform, Multispectral image, Algal phenomics

## Abstract

**Background:**

*Pyropia* is an economically advantageous genus of red macroalgae, which has been cultivated in the coastal areas of East Asia for over 300 years. Realizing estimation of macroalgae biomass in a high-throughput way would great benefit their cultivation management and research on breeding and phenomics. However, the conventional method is labour-intensive, time-consuming, manually destructive, and prone to human error. Nowadays, high-throughput phenotyping using unmanned aerial vehicle (UAV)-based spectral imaging is widely used for terrestrial crops, grassland, and forest, but no such application in marine aquaculture has been reported.

**Results:**

In this study, multispectral images of cultivated *Pyropia yezoensis* were taken using a UAV system in the north of Haizhou Bay in the midwestern coast of Yellow Sea. The exposure period of *P. yezoensis* was utilized to prevent the significant shielding effect of seawater on the reflectance spectrum. The vegetation indices of normalized difference vegetation index (NDVI), ratio vegetation index (RVI), difference vegetation index (DVI) and normalized difference of red edge (NDRE) were derived and indicated no significant difference between the time that *P. yezoensis* was completely exposed to the air and 1 h later. The regression models of the vegetation indices and *P. yezoensis* biomass per unit area were established and validated. The quadratic model of DVI (Biomass = − 5.550DVI^2^ + 105.410DVI + 7.530) showed more accuracy than the other index or indices combination, with the highest coefficient of determination (R^2^), root mean square error (RMSE), and relative estimated accuracy (Ac) values of 0.925, 8.06, and 74.93%, respectively. The regression model was further validated by consistently predicting the biomass with a high R^2^ value of 0.918, RMSE of 8.80, and Ac of 82.25%.

**Conclusions:**

This study suggests that the biomass of *Pyropia* can be effectively estimated using UAV-based spectral imaging with high accuracy and consistency. It also implied that multispectral aerial imaging is potential to assist digital management and phenomics research on cultivated macroalgae in a high-throughput way.

## Background

Macroalgae contributes to around 10% of total global marine primary productivity, and its aquaculture production constitutes approximately 28% of total world marine aquaculture production by weight [12, 43]. Macroalgae production is economically important for providing food, medicine, cosmetics, and biofuel [13, 14]. The red macroalgae genus *Pyropia* (common name nori or laver), has been cultivated and consumed in East and Southeast Asia for over 300 years [3, 14, 30]. Up to 2.56 million tonnes of *Pyropia* (fresh weight) were harvested in 2017 and valued at approximately US$2.32 billion (FAO [12]. Fisheries and Aquaculture Information and Statistics Branch 2019). In comparison to other cultivated macroalgae, *Pyropia* has the highest commercial value per unit mass at $905 per tonne. It has high nutritional value, in particularly high protein content at ~ 25–30% of the blade dry weight and a delicious flavour [3, 12].

High-throughput phenotyping has been increasingly used in recent years in research on phenomics and breeding, as well as for the digital management of precision agriculture [1, 17, 49]. Among various measurable agronomic traits, biomass is the most basic, not only for evaluating the growth trend and estimating yield, but also for assessing the ecosystem services of vegetation [32, 38]. However, the conventional estimation of biomass is labour-intensive, time-consuming, manually destructive with a tendency to produce human error, and cannot provide data on large scale [8, 26]. Using unmanned aerial vehicle (UAV)-based multispectral or hyperspectral imaging techniques makes high-throughput phenotyping more efficient, accurate, and precise, as well as being non-destructive. It has been widely used in terrestrial agricultural research and management [42, 46, 57]. Using this technique, spatial and temporal crop biomass data can be obtained in time and can be used to analyse crop responses to dynamic environment conditions [28, 34]. Several studies have used this technique to estimate the biomass of crops such as maize [19, 56], wheat [5, 35], rice [9, 52], barley [2, 40], soybean [54, 60], and rapeseed [16, 34].

Comparatively, the marine cultured macroalgae have been less studied than terrestrial plants [37]. Until now, it has no such research been published on high-throughput phenotyping of cultivated macroalgae using UAV-based spectral imaging. Besides the social and economic factors, it might be due to the more complex environmental conditions of coastal area where mariculture is carried out, besides resuspended sediments, and coloured dissolved organic matter from terrestrial runoff, which not only limit the survey range but also interfere the imaging quality [21, 51]. Seawater can strongly absorb light in the red–near infrared (NIR) wavelength and reflects blue and green light, which in turn interferes with the spectral signal and influences the accuracy of imaging [20, 45]. However, for macroalgae floating on the seawater surface, especially for harmful macroalgae bloom, several studies have proved that it is feasible to estimate the biomass of *Ulva prolifera*, *Sargassum natans,* and *Trichodesmium* spp. based on spectral images [10, 21, 51]. Moreover, the benthic macroalgae such as *Codium tomentosum*, *Laminaria saccharina*, *Corallina officinalis* could be qualitative mapped by hyperspectral remote sensing in the coastal areas [6–8]. Therefore, theoretically, it is feasible to use UAV-based multispectral platform to estimate the biomass of cultivated macroalgae.

**Fig. 1:**
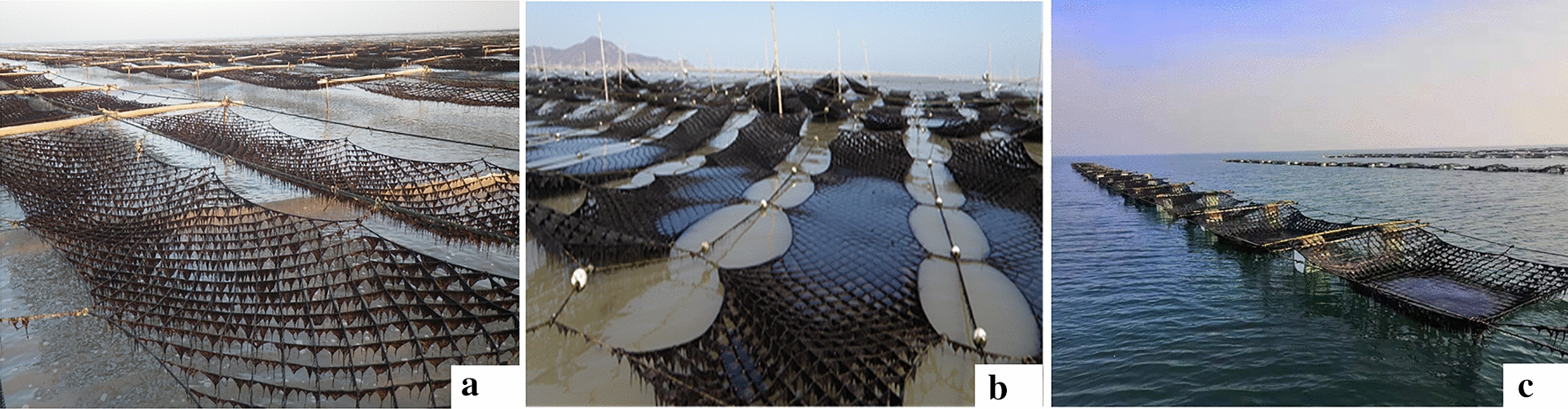
Three different cultivation methods of *Pyropia* cultivation. **a** Semifloating nets on the intertidal zone; **b** Nets on fixed pillars in the shoal region; **c** Turnover nets in the deep water zone

In the coast of Yellow Sea, *Pyropia* is cultivated on nets by three kinds of rafts: semi-floating, fixed pillars, or turnover (or full floating) (Fig. 1). The *Pyropia* nets are periodically exposed to the air during the low tide period, sometimes aided by artificial lifting, to follow the natural condition of *Pyropia* in the intertidal zone. This periodic exposure can decrease the epiphytes and competitors, and increases the protein content of *Pyropia* thallus [4, 29, 30]. Simultaneously, these specific exposure period can provide a time frame for piloting the high-throughput phenotyping using UAV-based spectral imaging on cultivated *Pyropia*.

In this study, *Pyropia yezoensis*, one of two widely cultivated *Pyropia* species, was investigated using UAV-based spectral imaging. The aim was to establish an algorithm model of biomass based on multispectral imaging data, which could be used for estimating *P. yezoensis* biomass in an accurate, high-throughput, and non-destructive way.

## Results

### Comparison of multispectral reflectance

**Fig. 2:**
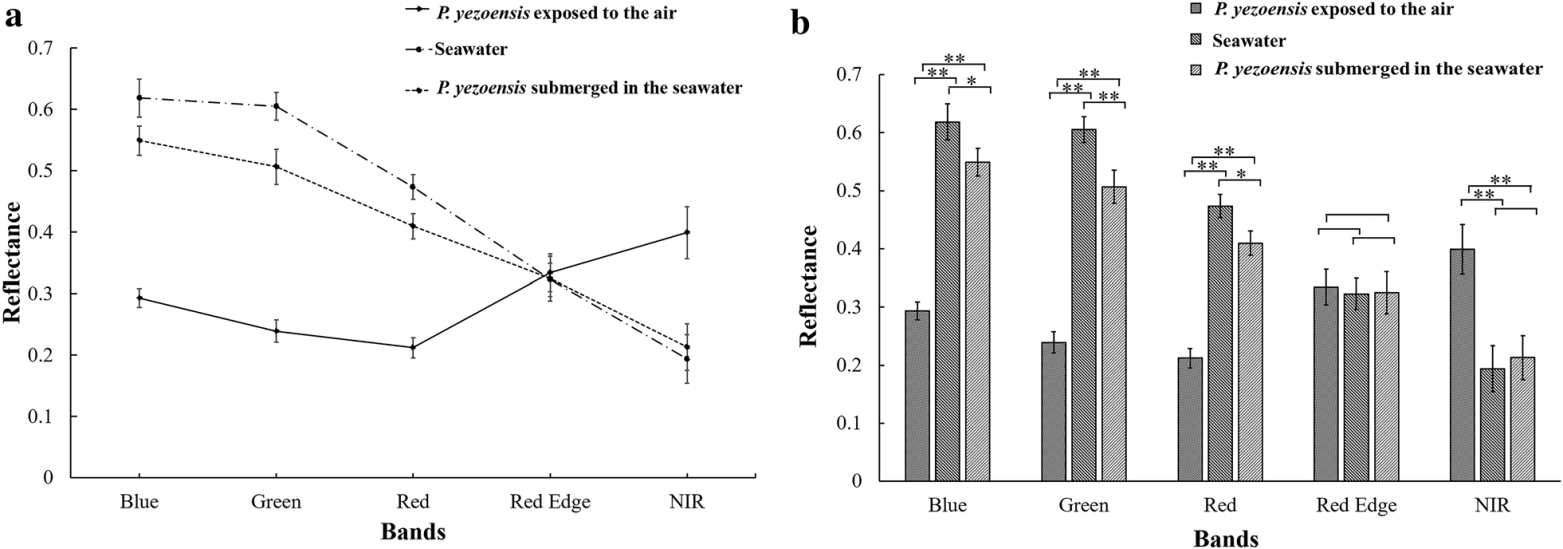
Multispectral reflectance at five wave bands (*P $< 0.05$; **P $< 0.01$)

The multispectral reflectance of *P. yezoensis* submerged in seawater was very similar to that of seawater, but quite different to that of *P. yezoensis* exposed to the air, which implied that seawater had a significant effect on seaweed (Fig. 2a). Compared with exposed *P. yezoensis*, the submerged *P. yezoensis* (floating just below the surface of the seawater) showed that the reflectance spectrum significantly increased in the blue, green, and red wavelengths (*P *< 0.01, Fig. 2b), and significantly decreased in the NIR wavelength (*P *< 0.01, Fig. 2b). On the other hand, compared to seawater, for submerged *P. yezoensis*, its red edge reflectance which should be raised by *P. yezoensis* had no significant difference with that of seawater (*P *> 0.05, Fig. 2b), neither did NIR reflectance (*P *> 0.05, Fig. 2b). Therefore, the period of *P. yezoensis* exposure to the air was more optimal for multispectral aerial imaging and was used in the followed measurements.

### Assessment of dehydrated effect

**Fig. 3:**
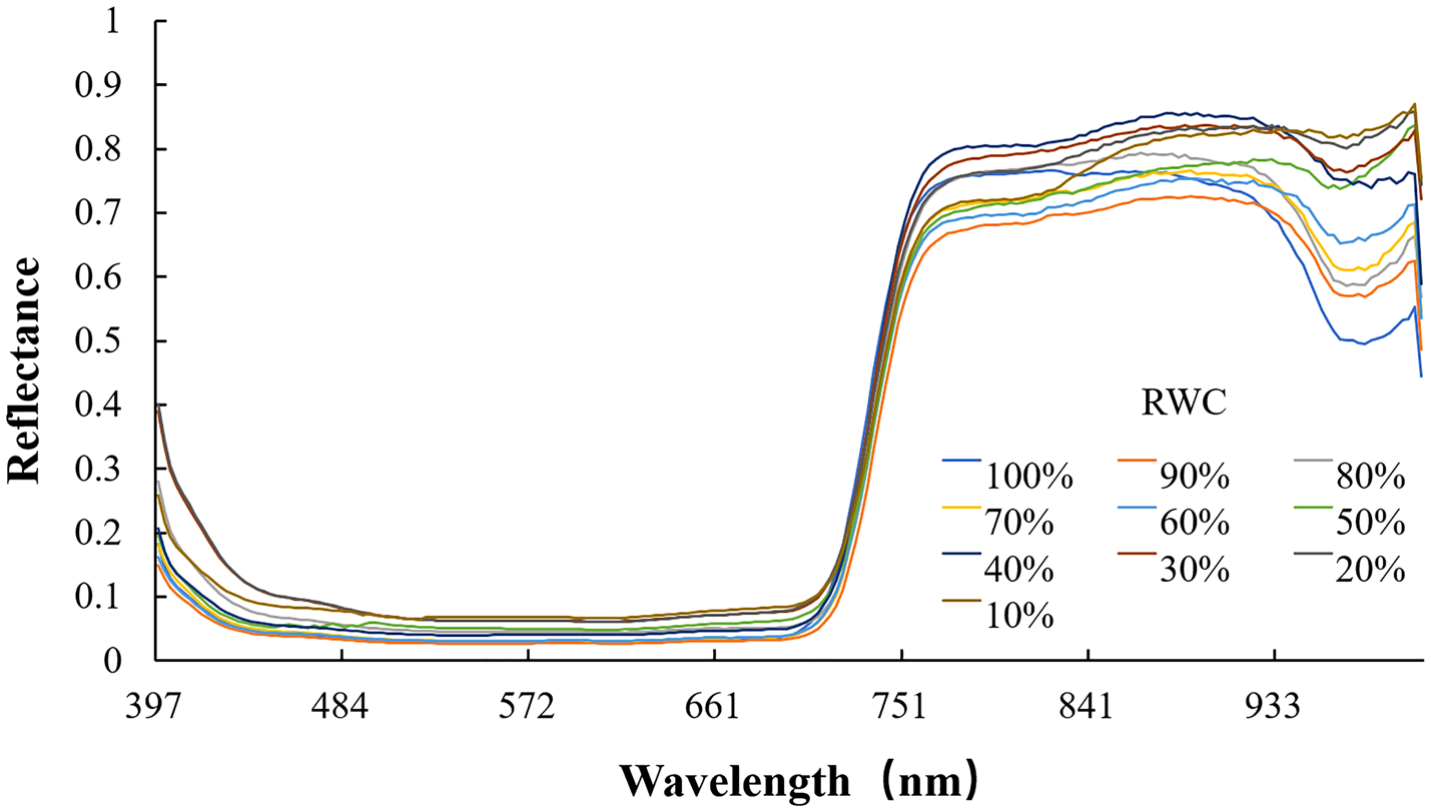
Hyperspectral reflectance of *P. yezoensis* under different RWC

**Fig. 4:**
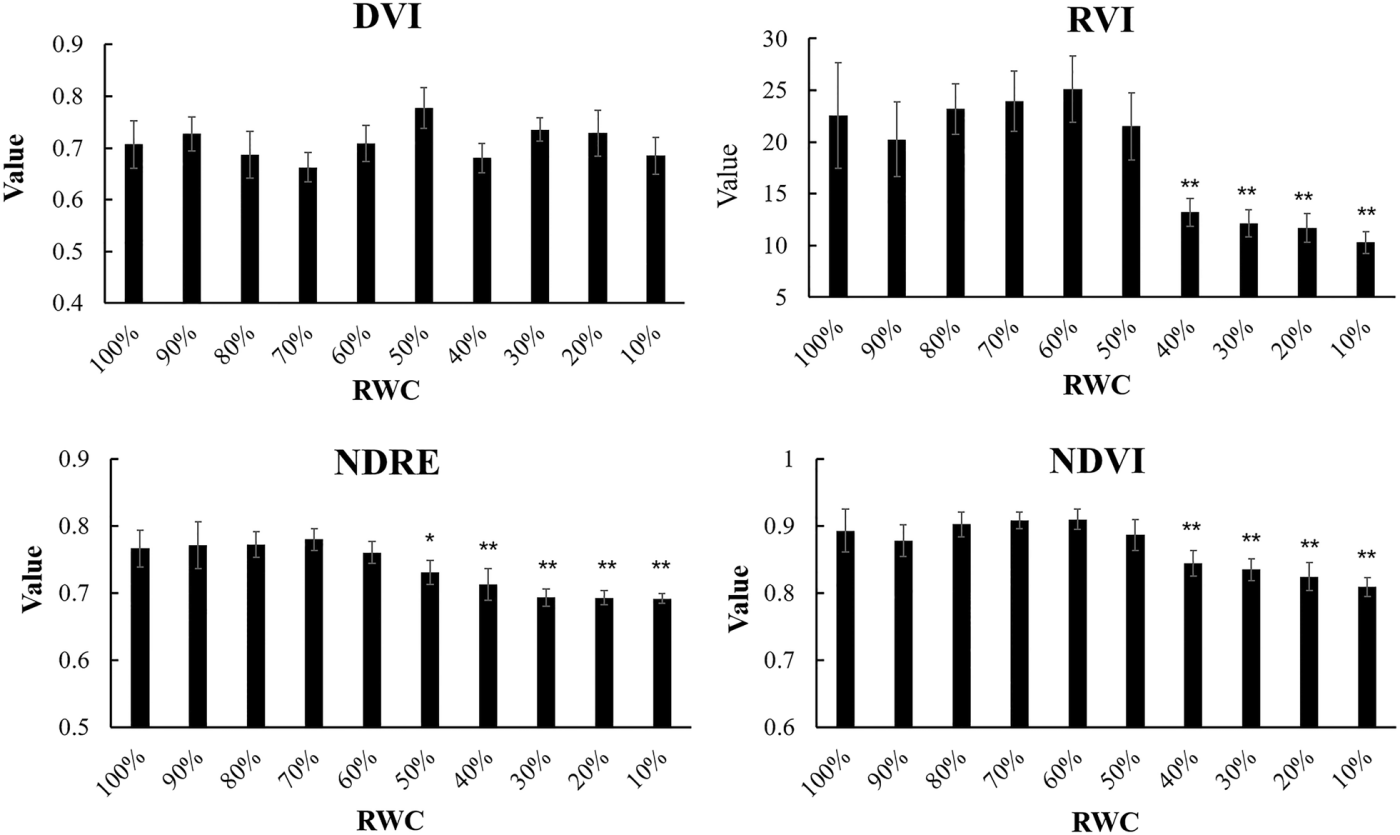
Vegetation indices of *P. yezoensis* under different RWC (*P $< 0.05$; **P $< 0.01$)

When exposed to the air, the water content of *P. yezoensis* thalli would gradually reduce and influence the reflection spectrum (Fig. 3). Laboratory experiments showed that the values of DVI, RVI, NDRE, and NDVI varied differently along with decreased relative water content (RWC) (Fig. 4). Compared with the control group (100% RWC), there was no significant difference in *P. yezoensis* from 90% RWC to 10% RWC in the values of DVI. However, there were significant decrease for RVI and NDVI when the RWC was under 40% (*P* $<$ 0.01), and relatively for NDRE under 50% (*P* $<$ 0.05). Generally, all four indices were relatively stable at RWC ranging from 100% to 60%.

**Fig. 5:**
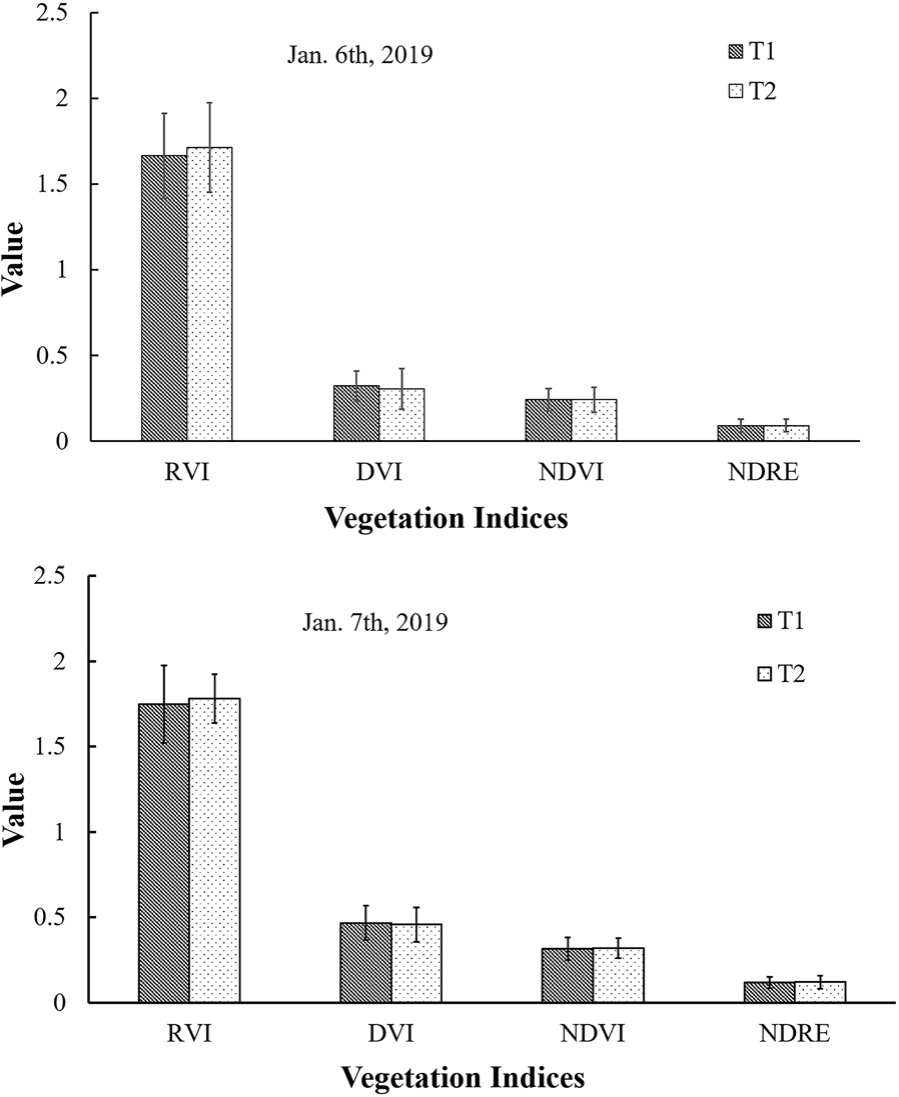
Comparison of the vegetation indices between differen*t* exposure time. T1, *P. yezoensis* once completely exposed to the air; T2, 1 h after T1

Further field experiments confirmed the suitable period for acquisition of aerial image data. The comparison between the time when *P. yezoensis* was completely exposed to the air and 1 h later showed that there was no significant change for the four vegetation indices (Fig. 5, *P *> 0.05), both on 6th and 7th January, 2019. This indicated that the spectral characters of *P. yezoensis* are stable during this period and suitable for data collection by aerial imaging.

### Biomass estimation models and accuracy assessment

**Fig. 6:**
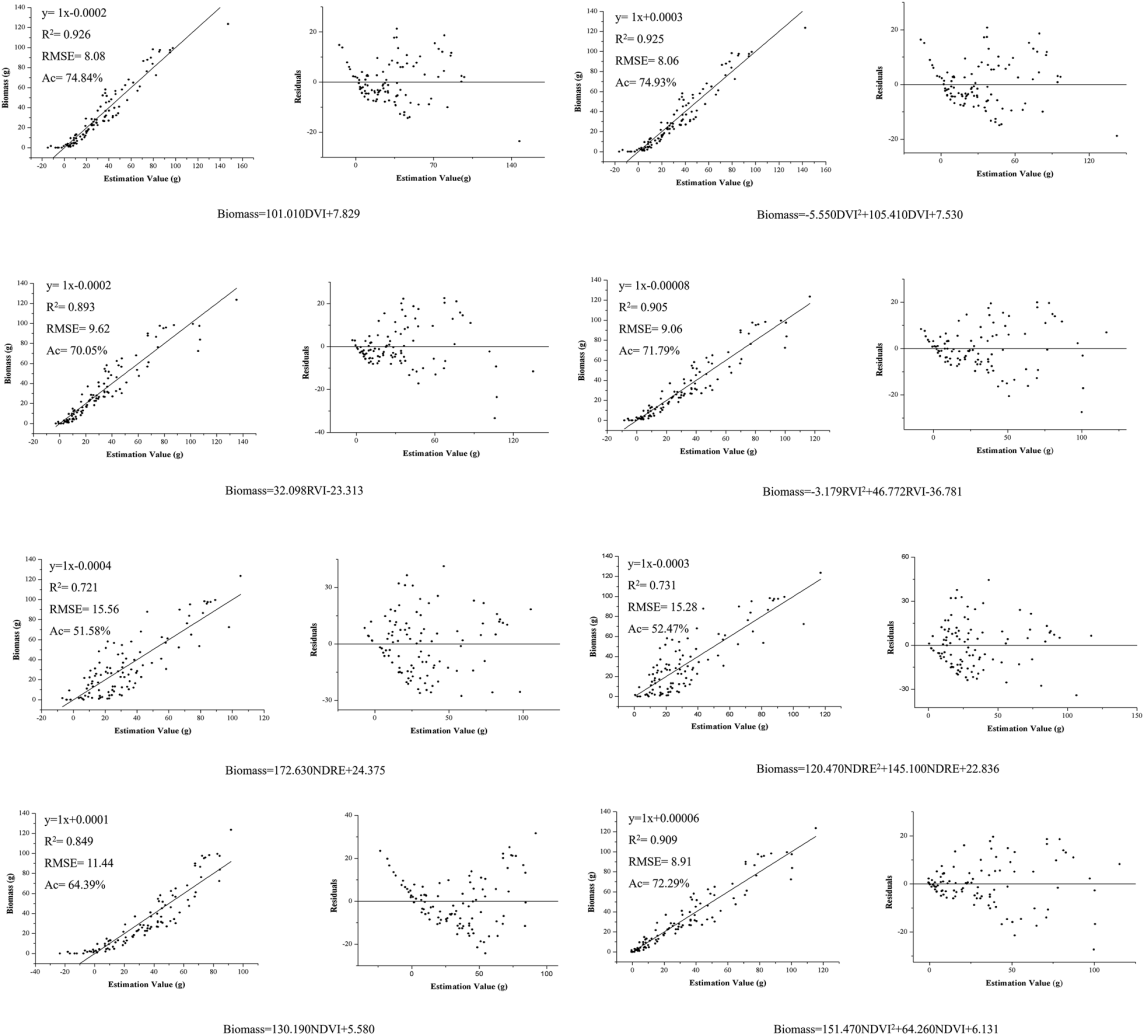
Regression models and residuals-analysis plots based on single vegetation index (n = 80)

**Fig. 7:**
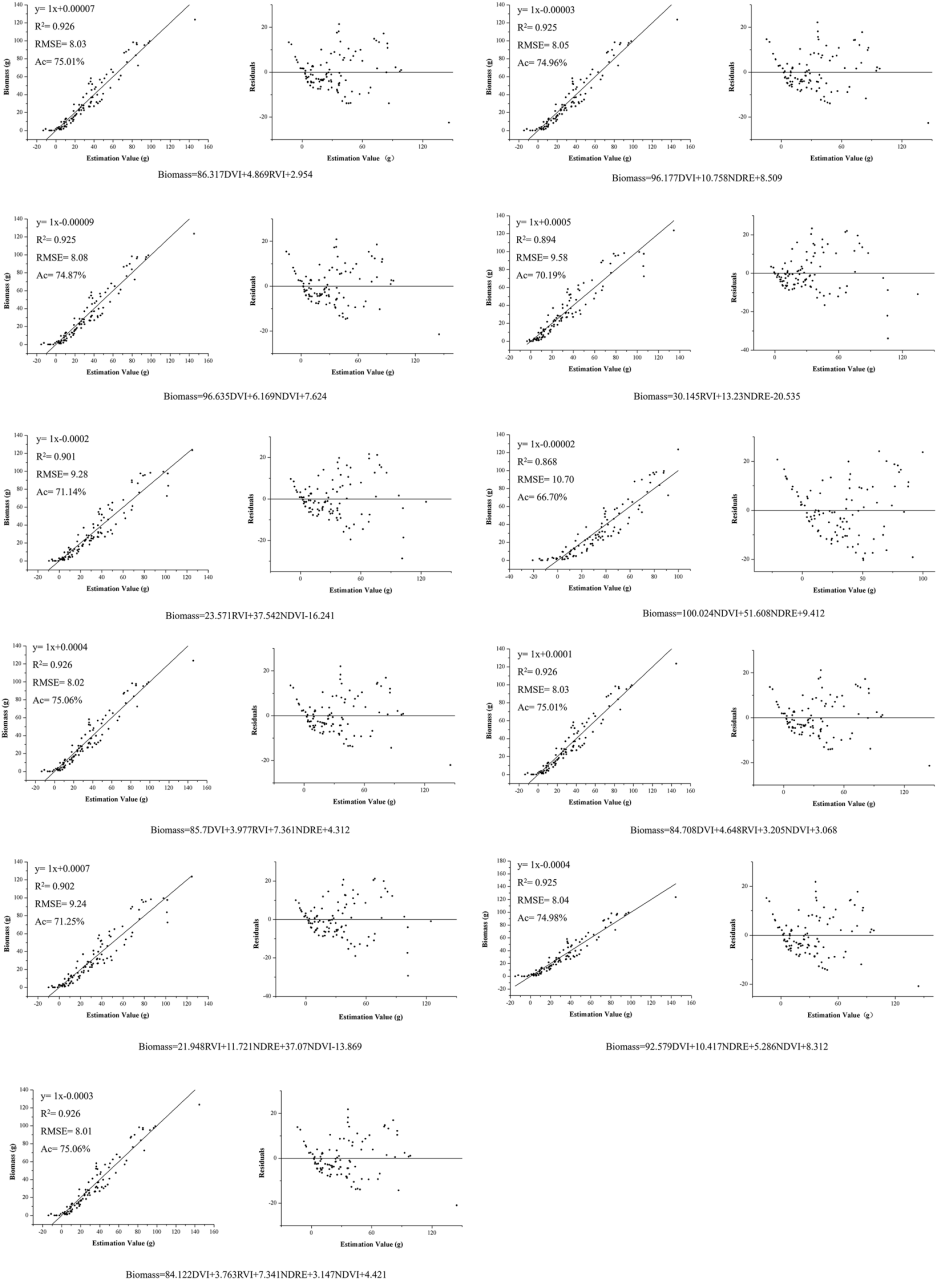
Regression models and their residuals-analysis plots based on combined vegetation indices (n = 80)

The strong and positive correlations existed between *P. yezoensis* biomass and individual vegetation index of DVI, RVI, NDVI, and NDRE, with correlation coefficients of 0.962, 0.945, 0.922, and 0.849, respectively (Table 1; n = 80, *P* < 0.01). This also confirmed that DVI, RVI, NDVI and NDRE were promising indicators for the biomass estimation. The calculation equations of biomass based on single or combined vegetation indices with their regression diagnostic plots of the predicted values and the distribution plots of residuals are shown in Figs. 6 and 7. For the single vegetation index, the quadratic model performed better than that of the linear regressions with higher R^2^, Ac and lower RMSE (Fig. 6). The optimal regression equation was the quadratic model of DVI (Biomass = − 5.550DVI^2^ + 105.410DVI + 7.530). Its R^2^, RMSE, and Ac were 0.925, 8.06, and 74.93%, respectively, and residual interval was between − 20.76 and 18.84 (Fig. 6). RVI and NDVI exhibited relatively high R^2^ of above 0.8, while NDRE showed relative lower R^2^ values of 0.721 and 0.731, respectively. For the combined vegetation indices, the regressions with vegetation indices of DVI and RVI had higher accuracy (R^2^ > 0.925, Ac > 75%) than that with combination with NDVI and NDRE. However, the optimal combination was that of DVI, RVI, NDRE, and NDVI (Biomass = 84.122DVI + 3.763RVI + 7.341NDRE + 3.147NDVI + 4.421), which values of R^2^, RMSE, and Ac were 0.926, 8.01, and 75.06%, respectively, and the residual interval was between − 21.78 and 21.02 (Fig. 7). Comparing the two optimal regression models based on single and combined indices respectively, although the later have little bit higher R^2^, Ac and lower RMSE, the residual interval of the former (quadratic model of DVI) was smaller, which indicated a more accurate estimation of biomass. And save for simplification, the quadratic model of DVI (Biomass = − 5.550DVI^2^ + 105.410DVI + 7.530) was considered more applicable for estimating the biomass of *P. yezoensis*.

**Table 1:** Correlation coefficient of biomass with each vegetation index (n = 80, *P* $< 0.01$)

	DVI	RVI	NDRE	NDVI
Biomass	0.962	0.945	0.849	0.922

### Model verification

**Fig. 8:**
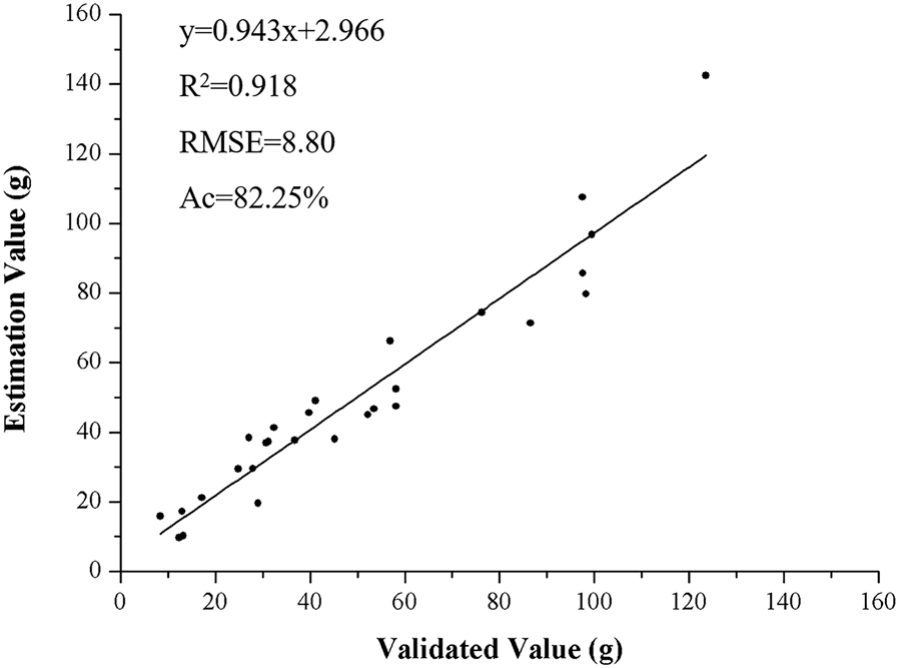
Relationship between the estimated and validated value of *P. yezoensis* biomass

As it shown in Fig. 8, the validated values of biomass compared reliably with the estimated values based on the optimal regression model (Biomass = − 5.550DVI^2^ + 105.410DVI + 7.530). The model consistently predicted the biomass with an R^2^ value of 0.918, RMSE of 8.80, and Ac of 82.25%. The slope of the regression lines between the estimated and validated values was 0.943, indicating a good prediction according to the criterion of Jamieson et al. [23].

## Discussion

In this study, UAV-based multispectral imaging was introduced to estimate the biomass of *Pyropia*, and the results have demonstrated its potential application in marine aquaculture. Although techniques and facilities used in *Pyropia* cultivation have improved in recent years, the long distance offshore makes it difficult to supervise the situation on a large scale. The UAV-based spectral imaging system established in this study can be used for monitoring of the spatial and temporal status of cultivated *Pyropia* at large scales in a more high-throughput and cost-saving way.

In spectral acquisition, the variations of coastal environment would cause uncertainties. Previous studies reported that the environmental factors, such as clouds and tidal stage of the coastal area during UAV flight could result in the radiometric variability [11, 19]. In this study, the multispectral images were collected under the similar sky conditions and the flight time was closed to solar noon [48]. In addition, the irradiance sensor loaded on the UAV platform could help rectify the difference of light conditions. Moreover, in coastal areas, seawater may influence the reflection values derived from the UAV-based spectral images. For instance, seawater can absorb in the red to NIR wavelengths and elevate the reflectance in the blue to green wavelengths [10, 21], especially in the coastal region with active river discharge and coastal turbid currents [31]. The Yellow Sea is specifically characterised by high turbidity, which would enhance the influence on spectral reflection [45]. Usually, the interference from different depths of seawater on spectral reflectance also limits the use of spectral imaging on most cultivated macroalgae, such as *Saccharina japonica* and *Gracilariopsis lemaneiformis,* which constantly submerged below the water surface throughout the culture period [24, 44, 53]. Compared to them, the cultivation of *P. yezoensis* involves periodic exposure out of the water which could be utilized for spectral imaging. In this study, it proved that the seawater significantly affected the reflection of *P. yezoensis*. Particularly, the seawater made it very difficult to distinguish between seawater and submerged *P. yezoensis* in the NIR wavelength, which is essential for calculating almost all vegetation indices. Therefore, at primary stage, multi-spectral imaging is suitable to be taken during the *Pyropia* exposure period. After solving the related techniques problems by developing higher resolution of the reflectance spectrum or eliminating disturbance from seawater, it could be feasible to use UAV-based spectral imaging for other cultivated macroalgae.

The vegetation index derived from spectral images has been widely used in the estimation of terrestrial crop biomass [35]. Same as terrestrial crops, the main pigment of the photosynthetic reaction centre of *Pyropia* is chlorophyll-a [59], which primarily absorbs the light of the red and blue wavelengths but scatters most of solar radiation in the NIR wavelengths [45]. To obtain plant traits including biomass using spectral techniques, the reflectance of red and NIR wavelengths have been commonly used to calculate vegetation indices such as DVI, NDVI, and RVI [9, 16, 20, 28].

However, it showed that the spectral reflectance can be influenced by plant water stress [18, 36, 39, 58]. Most *Pyropia* species lose 85–95% of cellular water during daytime low tide [4]. This dehydration would also affect the spectral characters of *P. yezoensis* and hence influence the biomass estimation. In this study, through laboratory and field experiments, we defined a stable time frame for collecting the UAV-based spectral imaging, which was within 1 h after *P. yezoensis* exposed to the air. The results showed that no effects on the values of DVI, RVI, NDVI, or NDRE. This time frame ensured the application of UAV-based spectral imaging for *Pyropia* biomass estimation using the four indices.

In this study, using UAV based five spectral band sensors, 4 existing vegetation indices were derived instead of creating a new index from specific spectral wavelengths. It was corroborated its convenience, effectiveness and relatively lower-costing by previous study [16]. Among the four indices, the DVI were finally selected for biomass estimation models, which based on the reflectance of NIR and red spectral wavelength. It showed that the DVI was highly significantly correlated with biomass and present more stable than other index in the laboratory measurement on different dehydration level of *Pyropia*. In the study on biomass estimation of *Spartina alterniflora*, Zhou et al. [61] also proved that the quadratic regression model of the DVI was more suitable than other vegetation indices. Moreover, in the present study, the regression model of Biomass = − 5.550DVI^2^ + 105.410DVI + 7.530 shows high accuracy on biomass estimation of *Pyropia*. It is the first success in biomass estimation on cultivated macroalgae using UAV-based multi-spectral imaging, which suggests that there is high potential to establish efficient, accurate, and high-throughput phenotyping for mariculture.

On the other hand, red and brown macroalgae have more pigments besides chlorophyll-a, which could be characterized wavelength-specific absorbance and reflectance properties and used for high-throughput phenotyping [32]. Hu et al. [21] reported that the phycourobilin and phycoerythrobilin, which were also the dominant pigments of *Pyropia*, resulted in spectral curvatures between 469 and 555 nm using hyperspectral satellite. However, in this study, we did not discover the similar phenomenon, which might be limited by the broad band width of the multispectral sensor. Therefore, if the UAV platform is equipped with a hyperspectral sensor, more spectral features might be used for more accurate estimation [55]. In fact, in our laboratory experiment on dehydration effect, the continuous spectrum with high resolution presented detailed information among different dehydration levels of *P. yezoensis*. After exploring more specific indices for different phenotraits under controllable conditions in the lab, it could be expected to utilise UAV equipped with a hyperspectral sensor for high-throughput phenotyping of more traits and more cultured macroalgae species in the field. The high-throughput acquisition of more morphological and physiological phenotraits of macroalgae would contribute to the phenomics study of the interactions between the genome and the environment.

Moreover, a UAV-based spectral imaging system can be utilised in ecosystem-based management by providing prompt and instinctive information on large-scale monitoring. Several studies reported that large-scale cultivation of macroalgae benefited the coastal environment by extracting inorganic nutrients (such as nitrogen, phosphorus, and carbon dioxide), mitigating adverse environmental impacts, and reducing the occurrence of harmful algal blooms [14, 30, 41, 53]. The UAV-based spectral imaging systems could help to reasonably manage cultivated area, and predict algal blooms [25]. And after further validation on larger scale and a long term of years, the biomass estimation model established in this study will be benefit the cultivation management of *P. yezoensis* for sustainable aquaculture.

## Conclusions

This study established a regression model using a vegetation index (DVI) and a feasible method to estimate the biomass of *Pyropia* using UAV-based multispectral imaging. High accuracy of the estimated model was validated by the strong similarities between estimated and manually measured biomass. Compared with the conventional measurement, the model could monitor the spatial and temporal status of cultivated *Pyropia* in a large-scale and cost-saving manner.

## Methods

### Experimental sites

**Fig. 9:**
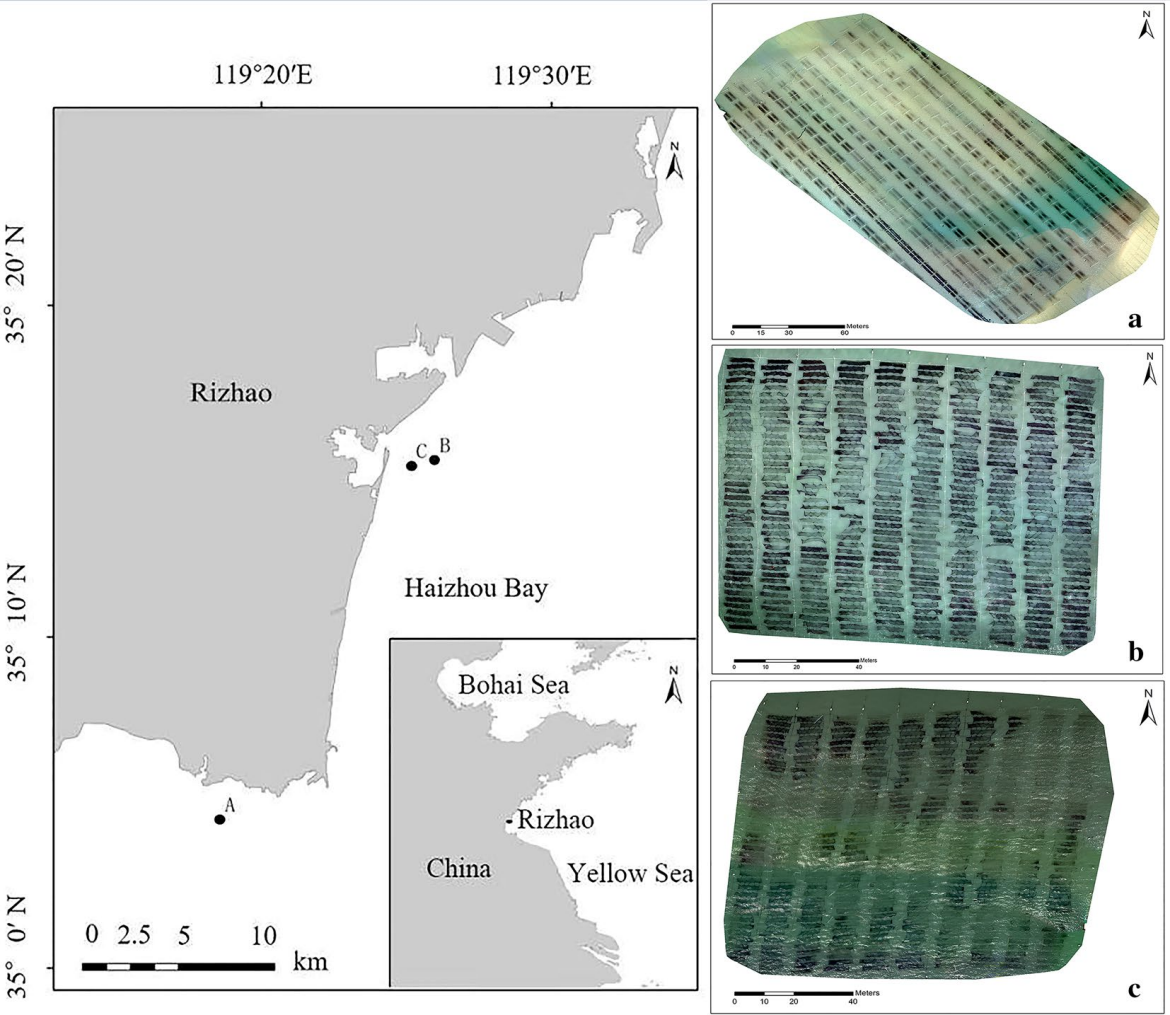
Study sites with RGB images of plot A, B and C

This study was conducted in the north of Haizhou Bay, in the midwestern coast of the Yellow Sea. Haizhou Bay, with 25 km of winding coastline, experiences regular semidiurnal tide. The tidal cycle is approximately 12 h 18 min and the average tidal range is approximately 344 cm. In this area, the aquaculture area of *P. yezoensis* was more than 200 hectares in 2017 [33]. The study area consisted of three parts, plots A, B, and C (Fig. 9). The cultivation rafts of plot A was semi-floating in the intertidal mudflat, and those of plots B and C were fixed pillars in shallow sea.

### Image data acquisition

**Fig. 10:**
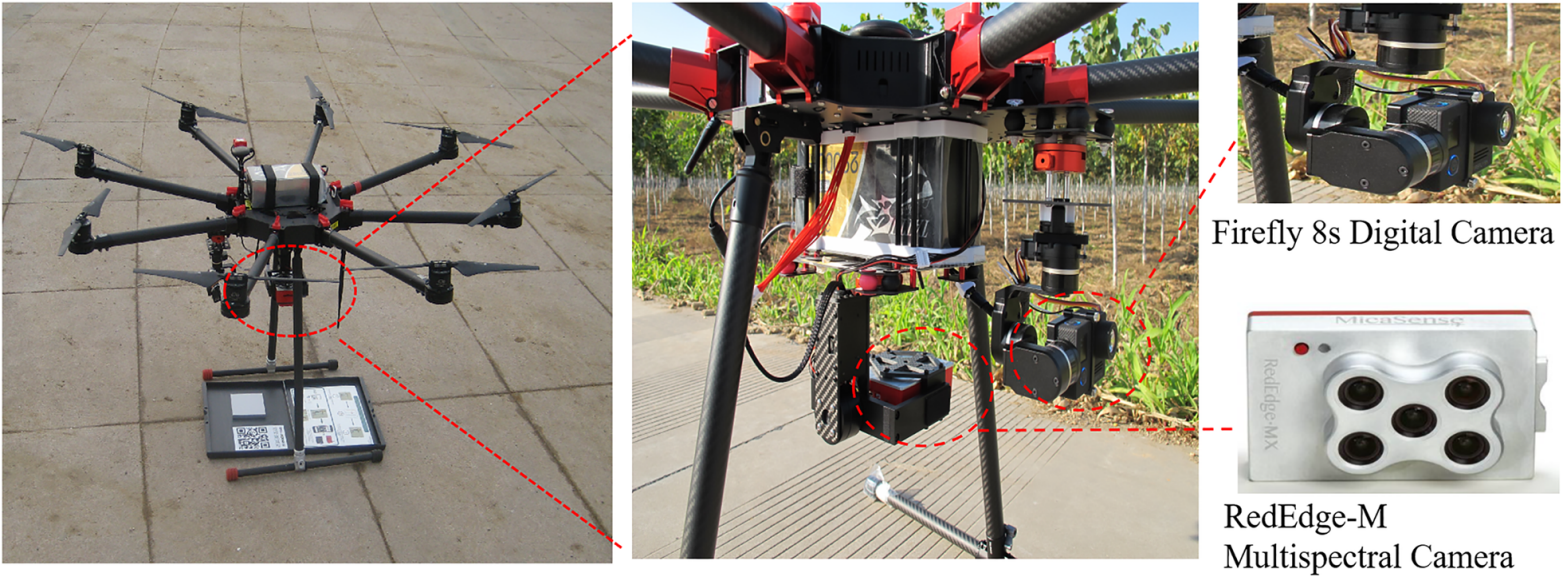
UAV platform and sensors

Airborne multispectral and digital images of the study areas were acquired using a RedEdge-M sensor (MicaSense, USA) and a Firefly 8 s camera (Hawkeye, China), respectively (Fig. 10). The RedEdge-M sensor is comprised of a solid state with five spectral bands ranging from 400 nm to 900 nm. The wavelengths of each band were blue (475 nm centre, 20 nm bandwidth), green (560 nm centre, 20 nm bandwidth), red (668 nm centre, 10 nm bandwidth), red edge (717 nm centre, 10 nm bandwidth), and NIR (840 nm centre, 40 nm bandwidth). The resolution of the sensor was 1280 × 960 pixels with a field of view of 47.2°. Both sensors were flown onboard an Ecodrone UAS-8 Multifunctional UAV (Ecotech Ecological Technology Ltd., China). The flights altitude were approximately 40 m above cultivated *Pyropia* (Table 2). The radiometric calibration images of the RedEdge-M sensor were captured on a calibrated reflectance panel (MicaSense, USA) before each flight.

**Table 2:** Details of the flight in this study

Region	A	B	C
Flight date	6th and 7th Jan, 2019	22nd Nov, 2018 and 13th Jan, 2019	13th Jan, 2019
Area (m^2^)	25200	13150	12800
Flight time (min)	35	20	20
Flight height (m)	40	40	40
Line spacing (m)	10	10	10
Forward overlap	Average overlap > 70% (Timer exposure mode)	Average overlap > 70% (Timer exposure mode)	Average overlap > 70% (Timer exposure mode)
Side overlap	70%	70%	70%
Resolution	1280 × 960	1280 × 960	1280 × 960

To investigate the multispectral reflectance characters of *P. yezoensis* when it exposed to the air or submerged in seawater,, the UAV imaging was taken when ten nets were randomly selected by lifting them out of the water, in plot B on 22nd November 2018 (Table 2). Each 100 pixels (0.6 × 0.6 m) were extracted from images of exposed *P. yezoensis*, submerged *P. yezoensis* and seawater for comparing their multispectral reflectance. To check the potential influence of dehydration on reflectance, a laboratory experiment was conducted on *P. yezoensis* with a series of relative water content (RWC). The *P. yezoensis* thalli were spread flat on a plate to obtain the RWC from 100% to 10% in a gradient of 10% at 10 °C room temperature following the methods of Sun et al. [47]. The spectrums of *P. yezoensis* were measured using the hyperspectral camera Specim IQ (Specim, Finland) in 400–1000 nm range. And in the field, extra experiments were carried out to decide a suitable aerial imaging time. In plot A during the low tide period, the UAV-boarded multispectral sensors were flown twice at the time of *P. yezoensis* once totally exposed to air and 1 h later. The 100 pixels (0.6 × 0.6 m) were selected and compared their derived vegetation indices based on multispectral reflectance. Consistency in the investigated sites was ensured by determining the same global positioning system (GPS) coordinates using a Garmin 12 channel GPS receiver (Garmin, Taiwan). The field experiments were conducted twice on 6th and 7th January, 2019, respectively (Table 2).

### Data processing

**Fig. 11:**
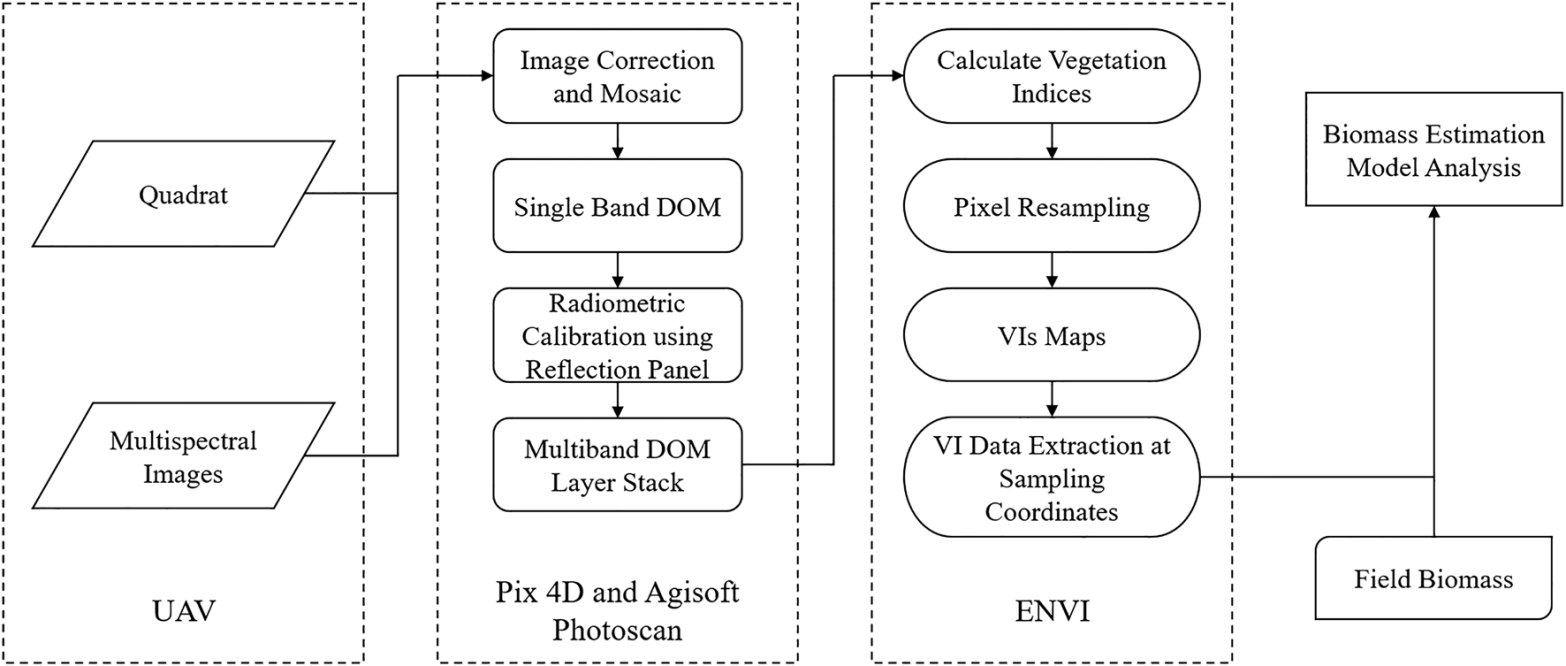
Schematic workflow of model establishment

Data processing was conducted as shown in Fig. 11. The images were jointed and orthorectification was taken using Pix4D 4.1.2 (Lausanne, Switzerland) and Agisoft Photoscan software. They were then subjected to geometric correction processing using the measurements of the 12 ground control points. After geometric correction, the radiometric calibrations were performed using Pix4D software. Radiometric calibration was carried out using the calibration images of the reflectance panel with known reflectance values. Each time before the UAV platform took off to the object regions, the multispectral camera acquired the images of the reflectance panel in advance. Using the corresponding values of the calibrated reflectance panel, the captured images data were carried out the radiometric correction using Pix4D or Photoscan automatically. Radiometric corrections were used to improve radiometric data quality and correct the spectral reflectance from images. The images acquired by the UAV-based multispectral sensors were performed using ENVI 5.1 software. To filter out the noise formed by the seawater background, kernel neighbourhood maximal calculation was performed. Resampling was used to control the number of pixel points involved in computation.

The reflectance values were derived using ENVI 5.1 software. The four vegetation indices, the difference vegetation index (DVI), ratio vegetation index (RVI), normalised difference of red edge (NDRE), and normalised difference vegetation index (NDVI) were calculated following the equations shown in Table 3 in ENVI 5.1.

**Table 3:** Vegetation indices used in this study

Vegetation Index	Formula	Reference
RVI	$\rho_{NIR} /\rho_{R}$	[27]
DVI	$\rho_{NIR}$-$\rho_{R}$	[50]
NDRE	$\left( {\rho_{NIR} - \rho_{RE} } \right)/\left( {\rho_{NIR} + \rho_{RE} } \right)$	[15]
NDVI	$\left( {\rho_{NIR} - \rho_{R} } \right)/\left( {\rho_{NIR} + \rho_{R} } \right)$	[22]

In the equations of Table 3, $\rho_{NIR}$, $\rho_{R} ,$ and $\rho_{RE}$ are the measured reflectance of NIR, red, and red edge bands, respectively.

The reflectance of *P. yezoensis* measured by the Specim IQ hyperspectral camera was transformed to RedEdge-M equivalent reflectance using the spectral response function of the RedEdge-M sensor, as the following equation.

1 $$R_{\text{RedEdge-M}} \left( {\lambda_{i} } \right) = \frac{{\smallint F_{0} \left( \lambda \right)S_{i} \left( \lambda \right)R\left( \lambda \right)d\lambda }}{{\smallint F_{0} \left( \lambda \right)S_{i} \left( \lambda \right)d\lambda }}$$

In the equation above, the *S*_*i*_ (λ) is the spectral response function of the *i*-th band of the RedEdge-M, *F*_*0*_ (λ) is the average solar irradiance, *R* (λ) is the measured reflectance by the hyperspectral camera, and $R_{\text{RedEdge-M}}$(λ) is the RedEdge-equivalent reflectance. The processes were performed using ENVI 5.1.

### Field measurement of biomass

**Fig. 12:**
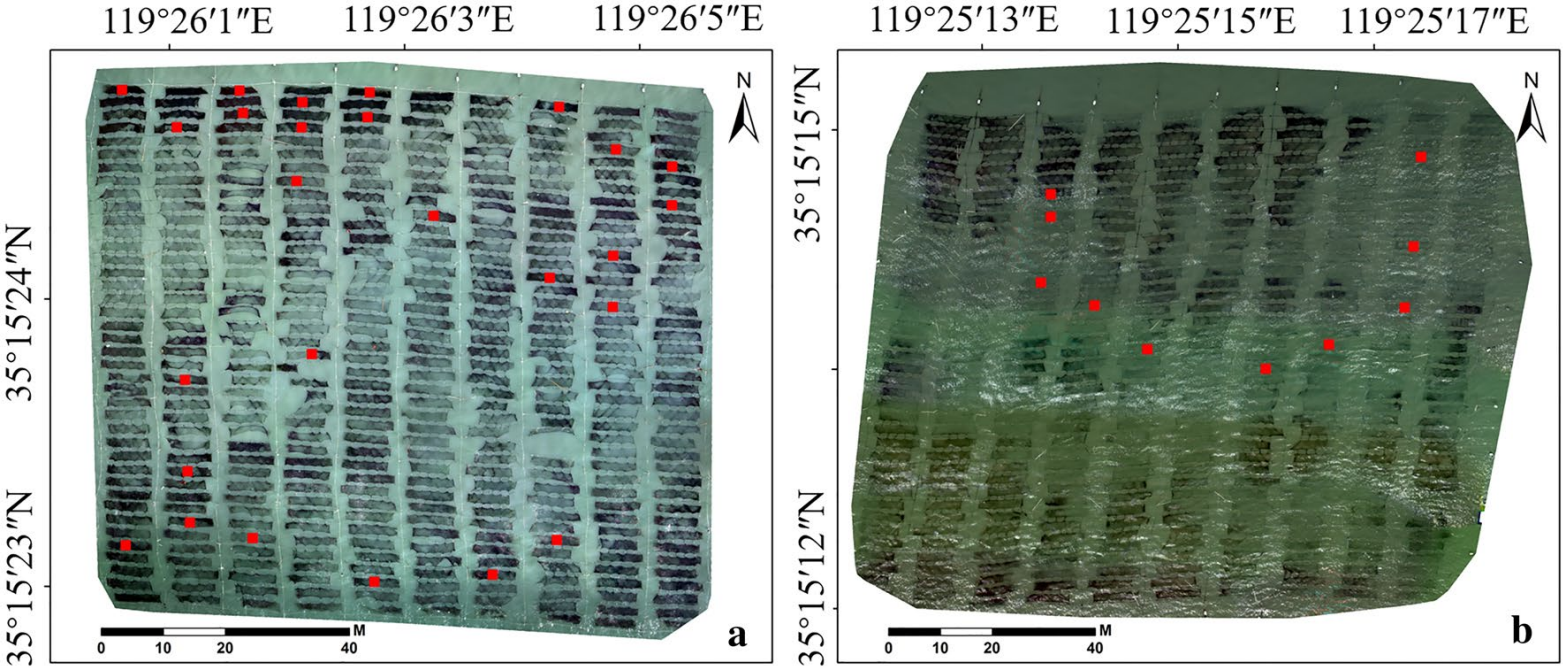
Sampling points in the study plot B (**a**) and C (**b**)

Matching the acquisition time of UAV multispectral imaging data, the field samples were synchronously obtained in plots B and C on 13 January 2019 (Table 2). Thirty-six *P. yezoensis* nets were randomly selected in plots B and C (Fig. 12). Each net was sampled by three 0.6 × 0.6 m quadrats within 1 h after *P. yezoensis* was completely exposed to the air. The locations of all sample quadrats were determined using the Garmin 12 channel GPS receiver. The *P. yezoensis* biomass of each quadrat was acquired by weighing the constant weight after drying in a heat oven (BPG-9070A) at 80 °C [5].

### Estimation model establishment and assessment

The regression of vegetation indices provides a simple and effective method for estimating biomass. In this study, the four vegetation indices were calculated from multispectral images using the mean reflectance values of a 0.6 × 0.6 m pixel in accordance with the same field sampling site (as determined using GPS coordinates). Eighty field quadrats out of a total of 108 were selected randomly as the training data for the establishment of the linear or non-linear regression model, and the remaining 28 quadrats were used for model validation. The simple linear or square regressions were used to estimate the parameters of calculation equations based on single or combined vegetation indices. The accuracy of each model was assessed using the root mean square error (RMSE), relative estimated accuracy (Ac) and coefficient of determination (R^2^). The smaller the RMSE value, the better the accuracy. Furthermore, the higher Ac and R^2^ values indicate greater similarities between the estimated and true values. The equations for these parameters are as follows:

2 $${\text{R}}^{ 2} = { 1} - \mathop \sum \limits_{i = 1}^{n} \left( {X_{i} - Y_{i} } \right)^{2} /\mathop \sum \limits_{i = 1}^{n} \left( {X_{i} - \bar{X}} \right)^{2}$$

3 $${\text{RMSE }} = \, \sqrt {\mathop \sum \limits_{i = 1}^{n} \left( {X_{i} - Y_{i} } \right)^{2} /n}$$

4 $${\text{Ac }} = \, (1 - {\text{RMSE}}/\bar{X}) \times 100\%$$

In the equations above, *X* and *Y* are the measured and estimated biomass values of sample *i, n* is the number of samples, and $\bar{X}$ is the average value of total measured biomass. The regression diagnostic plots of the different vegetation indices and the distribution of the predicted values and residuals were used for model assessment.

## Data Availability

The datasets analysed during the current study are available from the corresponding author on reasonable request.

## Abbreviations

UAV: Unmanned aerial vehicle
RWC: Relative water content
VI: Vegetation index
NIR: Near infrared
NDVI: Normalised difference vegetation index
RVI: Ratio vegetation index
DVI: Difference vegetation index
NDRE: Normalised difference of red edge
R^2^: Coefficient of determination
RMSE: Root mean square error
Ac: Relative estimated accuracy
ENVI: Environment for Visualising Images
GPS: Global Position System
DOM: Digital orthoimage
